# The role of thyroid function in female and male infertility: a narrative review

**DOI:** 10.1007/s40618-022-01883-7

**Published:** 2022-08-09

**Authors:** R. Mazzilli, S. Medenica, A. M. Di Tommaso, G. Fabozzi, V. Zamponi, D. Cimadomo, L. Rienzi, F. M. Ubaldi, M. Watanabe, A. Faggiano, S. La Vignera, G. Defeudis

**Affiliations:** 1 Endocrinology Unit, Department of Clinical and Molecular Medicine, Sapienza University of Rome, Sant’ Andrea Hospital, Rome, Italy; 2 Department of Endocrinology, Internal Medicine Clinic, Clinical Center of Montenegro, School of Medicine, University of Montenegro, Podgorica, Montenegro; 3 Unit of Endocrinology and Diabetes, Department of Medicine, University Campus Bio-Medico di Roma, Rome, Italy; 4 Clinica Valle Giulia, GeneraLife IVF, Rome, Italy; 5 Department of Experimental Medicine, Sapienza University of Rome, Viale Regina Elena, 328, 00161 Rome, Italy; 6 Department of Clinical and Experimental Medicine, Policlinico “G. Rodolico, ” University of Catania, Catania, Italy

**Keywords:** Thyroid, Fertility, Assisted reproduction technologies—ART, In vitro fertilization—IVF, Thyroid autoantibody, Semen

## Abstract

**Purpose:**

We herein aimed to review the new insights into the impact of impaired thyroid function on male and female fertility, spacing from spontaneous pregnancy to ART, with the objective of providing an updated narrative revision of the literature.

**Methods:**

This narrative review was performed for all available prospective, retrospective and review articles, published up to 2021 in PubMed. Data were extracted from the text and from the tables of the manuscript.

**Results:**

Thyroid dysfunction is frequently associated with female infertility, whereas its link with male infertility is debated. Female wise, impaired function is detrimental to obstetric and fetal outcomes both in spontaneous pregnancies and in those achieved thanks to assisted reproduction technologies (ART). Furthermore, the reference range of TSH in natural pregnancy and ART procedures has recently become a matter of debate following recent reports in this field. On the other hand, the impact of thyroid function on the male reproductive system is less clear, although a possible role is suggested via modulation of Sertoli and Leydig cells function and spermatogenesis.

**Conclusion:**

Thyroid function should be carefully monitored in both male and female, in couples seeking spontaneous pregnancy as well as ART, as treatment is generally immediate and likely to improve chances of success.

## Introduction

Infertility, defined as the inability to conceive after at least 1 year of unprotected sexual intercourse, affects about 15% of couples, and it is particularly common in developing countries [1–3]. Male and female partners alone are responsible for 20–30% of cases, respectively, but contribute to 50% of cases overall [1].

There is a close link between thyroid function and female fertility: physiologically, pregnancy has a significant effect on the thyroid gland, and thyroid dysfunction has long been associated with female infertility [4] with both obstetric and fetal outcomes being well established [5]. Furthermore, the reference range of TSH in fertility, pregnancy, as well as in assisted reproduction technologies (ART) has become a matter of debate. On the other hand, the impact of thyroid function on the male reproductive system is debated, and a role for thyroid hormones in influencing Sertoli and Leydig cells as well as spermatogenesis has been proposed [6–8].

We herein aimed to review the new insights on the relationship between impaired thyroid function and male and female fertility, spacing from spontaneous pregnancy to ART, with the objective of providing an updated narrative revision of the literature.

## Materials and methods

This narrative review was performed for all available prospective, retrospective and review articles, published up to 2021 in PubMed. Data were extracted from the text and from the tables of the manuscript. The keyword search used included “thyroid and female fertility”, “thyroid and pregnancy”, “hypothyroidism and pregnancy”, “hyperthyroidism and pregnancy”, “thyroid autoimmunity and pregnancy”, “thyroid and recurrent pregnancy loss”, “thyroid and IVF”, “hypothyroidism and IVF”, “hyperthyroidism and IVF”, “thyroid autoimmunity and IVF”, “thyroid and miscarriage”, “thyroid and male fertility”, “thyroid and male infertility”, and “thyroid and semen.”

## Thyroid and female fertility

### Physiology

A well-functioning thyroid is crucial in pregnancy, and it undergoes physiologic changes to sustain fetal growth. There is a notable increase in thyroid gland size during pregnancy, by 10% in women who are well supplied in iodine and by to 20–40% in those who are iodine deficient [9]. The thyroid function changes in two ways: by increase in thyroxine binding globulin (TBG) due to estradiol level, and stimulatory effects of human chorionic gonadotropin (hCG), with repercussion on the hypothalamic–pituitary–thyroid axis [9].

An important role in the central and peripheral crosstalk is also played by adipokines; specifically, kisspeptin, which is essential for human reproduction acting on the hypothalamus and stimulating GnRH production, may also stimulate TSH [10]. Furthermore, leptin, which is produced by adipocytes and regulates food intake and energy storage, influences the hypothalamus–pituitary–thyroid axis by regulating the expression and stimulating thyrotropin-releasing hormone (TRH) [11, 12].

These conditions result in different thyroid-stimulating hormone (TSH) and free T4 (fT4) reference range than in the period out of gestation. In fact, TSH level decreases in the first trimester of pregnancy by 20–50%, due to hCG stimulatory effect on TSH receptor, leading to an fT4 increase in the same trimester, reaching maximum concentrations by 16 weeks of gestation, and consequently TSH increasing and fT4 lowering throughout the rest of gestation. In 15% of pregnant women during the first trimester, TSH level is below the lower limit of reference range of 0.4 mU/L [5]. In multiple pregnancies, it is expected that TSH level is even more suppressed due to higher hCG concentration [13]. Previous data proposed TSH upper reference limit of 2.5 mU/L in the first trimester and 3.0 mU/L in the second and third trimester [14]; recent studies proposed wider ranges, and societies now recommend using the reference range for each trimester adjusted for the population (local laboratory ranges), and T4 instead of fT4 as more specific for the pregnancy, although, as this is not readily available in all countries, many clinicians rely on TSH to monitor thyroid function throughout pregnancy [9] (Table 1). When population- and trimester-specific reference ranges for TSH are not available, an upper reference of approximately 4 mU/mL may be used [9].

**Table 1:** Reference limits of TSH and thyroid hormones dosage in pregnancy according to Guidelines

	2014 European thyroid association guidelines	2017 American thyroid association guidelines
TSH	- Trimester-specific reference ranges should be established in each laboratory. Local variations may occur - If not available, the following reference range are recommended: first trimester upper limits 2.5 mU/L; second trimester upper limits 3.0 mU/L; third trimester upper limits 3.5 mU/L	- Reference range for each trimester adjusted for the population Or - TSH < 4 mU/mL
fT4 T4	-T4 and fT4 assays are both suitable for thyroid function testing in pregnancy	-T4 analysis is suitable instead of fT4
fT3 T3	-	-T3 analysis could be helpful in the diagnosis and management of hyperthyroidism, in the presence of suppressed TSH

*TSH* thyroid-stimulating hormone, *fT4* free thyroxine, *fT3* free triiodothyronine

The importance of thyroid hormones in the female reproductive system has been highlighted since the evidence of TSH and thyroid hormone receptors (TR-a1 and TR-b1) on ovarian and oocytes surface [15], so its role in folliculogenesis, fertilization, embryogenesis, and in implantation, and maintaining pregnancy is inevitable. In this regard, in vitro studies suggest that thyroid hormones promote FSH-induced preantral follicle growth, activating the protein kinase B (Akt) pathway [16]. Furthermore, the expression of TSH receptor in human granulosa cells as well as the increase of cyclic adenosine monophosphate (cAMP) upon TSH stimulation have been described [17]. Consequently, thyroid hormones impairment could affect markers of ovarian reserve, including anti-Mullerian hormone (AMH) [18].

Thyroid hormones and hormone receptors also regulate the endometrium receptivity, which is the stage where all the actors, including thyroid hormones, cooperate to prepare and allow the implantation window of the blastocyst, with variations during the menstrual cycle [19, 20].

Moreover, alterations in thyroid hormones signaling could also have detrimental effects on the placenta, possibly even causing abortion; however, the molecular mechanisms involved have not been completely understood [21].

### Pathology

#### Thyrotoxicosis

The most common cause of thyrotoxicosis, a clinical syndrome resulting in exposure to thyroid hormone excess, is hyperthyroidism, which, in reproductive age, is usually due to autoimmune Graves’ disease (GD). GD occurs in 0.4–1.0% of women before pregnancy and about in 0.2% during pregnancy [15]. It is crucial to differentiate it from relatively common, hyperemesis gravidarum, which occurs in 0.3–1% of the cases. Other causes, such as toxic multinodular goiter and toxic adenoma, as well as subacute thyroiditis, are less common, and others are very rare. It is, therefore, important to distinguish these clinical manifestations to apply an adequate treatment.

Gestational transient thyrotoxicosis is more frequent than GD. A rare cause of hyperthyroidism in pregnancy is the mutation of the TSH receptor gene with functional hypersensitivity to hCG. Due to the stimulating effect of hCG on TSH receptor, serum TSH may decrease in the first trimester, with a peak of hCG between 7- and 11-weeks’ gestation. Even TSH levels lower than 0.1 mU/L may occur approximately in 5% of women by week 11 of pregnancy [9].

##### Impact on spontaneous conception

**Fig. 1:**
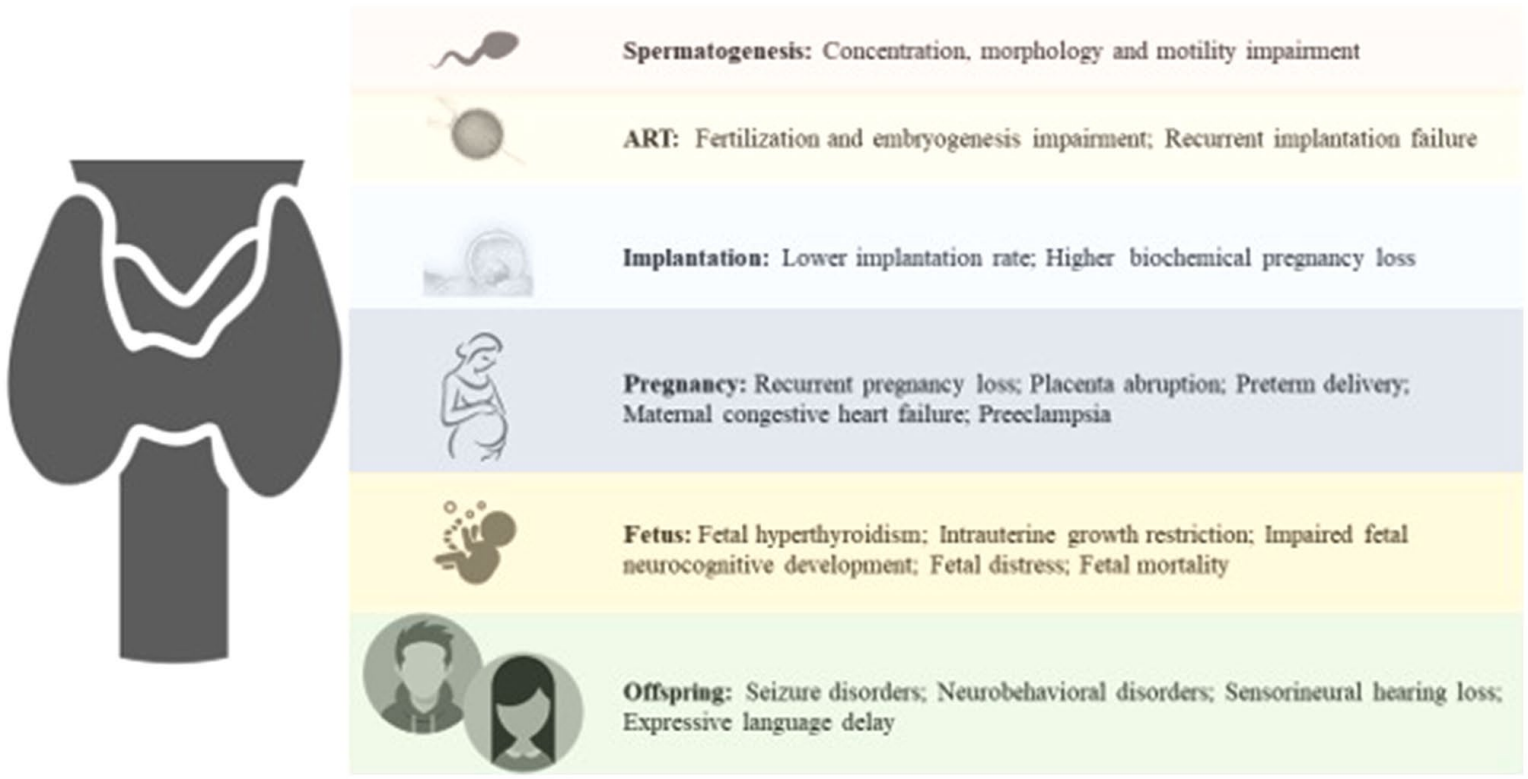
Impact of thyroid function on male and female reproductive system

Thyrotoxicosis results in increased serum levels of sex hormone binding globulin (SHBG) due to increase in estradiol levels, and a reduction of the metabolic clearance rate of estradiol. In women with hyperthyroidism, testosterone and androstenedione levels increase due to a higher production rate. Furthermore, the ratio of the conversion of androstenedione to estrone, as well as of testosterone to estradiol, increases [22]. These hormonal alterations result in menstrual cycle disturbances 2.5 times more frequent than in the general population [22] (Fig. 1).

Thyroid hormones increase hepatic SHBG production, which is also regulated by hepatocyte nuclear factor-4a (HNF-4a) in response to changes in the metabolic state of the liver [23]. Not only SHBG, but also other hormone binding proteins, such as corticosteroid-binding globulin (CGB) and thyroglobulin-binding-protein (TBG), are an index of thyroid action on the liver [24]. In severe hyperthyroidism, even symptomatic hypocortisolemia may be present [25]. In addition, prolonged hyperthyroidism leads to direct liver toxicity and hepatocyte anoxia with impaired liver function [26]. In patients with GD, LH secretion is increased compared to euthyroid patients [27].

Finally, a potential negative impact of RAI therapy on ovarian reserve has been reported [27].

##### Impact on ART outcomes

To date, no studies are available investigating the effect of hyperthyroidism on IVF outcomes. Probably, this is because patients with hyperthyroidism should postpone IVF techniques after normalization of thyroid function [27].

##### Impact on pregnancy and obstetric outcomes

Many maternal and neonatal complications rise due to the consequence of hyperthyroidism in pregnancy. Thyrotoxicosis can lead to significant maternal complications including miscarriage (defined as spontaneous pregnancy loss occurring before 20 weeks of gestation, although most miscarriages occur before 10 weeks of gestation [28]), recurrent pregnancy loss (defined as three or more consecutive miscarriages [28]), placenta abruption, preterm delivery, maternal congestive heart failure and preeclampsia [29]. Several studies suggest that fetal exposure to excessive levels of maternal thyroid hormone induces low birth weight, intrauterine growth restriction and stillbirth; furthermore, this condition could be related to seizure and neurobehavioral disorders in the offspring [30, 31].

#### Hypothyroidism

The prevalence of overt and subclinical hypothyroidism in the general population is about 0.3–0.4% and 4.3–8.5%, respectively, and it is mainly due autoimmune disease, thyroid surgery and effects of radiations or medications [9]. The prevalence in pregnancy is estimated at 0.3–0.5% and 2–3% (or even up to 5%), respectively [9]. The most frequent causes of hypothyroidism in pregnancy are the endemic iodine deficiency and the chronic autoimmune thyroiditis in iodine-repleted areas [32, 33]. If evidence linking overt hypothyroidism with infertility and poor pregnancy outcomes is straightforward, that regarding subclinical hypothyroidism is more controversial, and the reference range of TSH in fertility, pregnancy, as well as in ART has recently become a matter of debate.

##### Impact on spontaneous conception

In hypothyroidism, hormonal changes in androgens and estrogen have been observed, following lower metabolic clearance of androstenedione and estrone, and higher rate of peripheral aromatization. Although free fractions of testosterone and estradiol are increased, total hormone levels are decreased due to decreased SHBG concentrations, and prolactin may be increased along with TSH in response to increased hypothalamic thyrotropin-releasing hormone (TRH) [22]. As a consequence, about 80% of women with hypothyroidism present menstrual disturbances or irregularities [22]. Poor ovarian reserve (reflecting in follicle-stimulating hormone (FSH) typically > 14 IU/L on day 3 of menstrual cycle, antral follicle count < 5, and/or poor response to previous ovarian stimulation) was noticed with increasing TSH [34]. Among 239 women with infertility, those with unexplained cause had higher TSH levels, twice the number of them with TSH > 2.5 mIU/L compared to women with couple infertility due to a male factor [35].

##### Impact on ART outcomes

No study is available investigating the impact of overt hypothyroidism on IVF outcomes, as all patients are adequately treated prior to it. Different is for subclinical hypothyroidism: considering IVF/Intracytoplasmic sperm injection (ICSI) cycles, Fumarola et al. was the only author who found higher pregnancy rates in women with TSH levels ≤ 2.5 mIU/L compared to women with TSH levels > 2.5 mIU/L (22.3% vs 8.9%) [36]. These findings were not confirmed by the other authors, and most studies suggest similar pregnancy outcomes using a TSH cutoff level of < 2.5 mIU/L or one of < 4 mIU/L. To this regard, in a large retrospective cohort study including the first IVF cycle, no differences in clinical pregnancy, delivery or miscarriages were found using the two different cutoffs [37]. Chai et al. found similar live birth and miscarriage rates in patients undergone IVF/ICSI cycles, comparing a cutoff of 2.5 mIU/L to that of 4.5 mIU/L [38]. Finally, Unuane et al. conducted a retrospective study considering cumulative live birth delivery rates after 6 IVF/ICSI cycles and found no statistically significant differences using a TSH cutoff of 2.5 mIU/L or one of 5 mIU/L [39]. Several studies compared the impact of TSH level before IUI. In a study by Karmon et al., patients with preconception TSH between 2.5 and 4.9 mIU/L did not have worse clinical and obstetrical outcomes (lower live birth or spontaneous abortion) [40]. Unuane et al. and Tuncay et al. confirmed these results reporting no significant differences in live birth, pregnancy or miscarriage rate in subgroups according to TSH level (TSH 2.5–5 mIU/L compared to TSH < 2.5 mIU/L) [41, 42].

There is extreme heterogeneity across existing studies, with a variety of factors possibly influencing the findings. The main reason is that the definition of “normal thyroid function” and “subclinical hypothyroidism” has been changed during the time. Accordingly, the decision on whether to start treatment with levothyroxine or not, due to TSH cutoff definition, may differ. The definitions or criteria of final outcomes in different studies were often unclear or missing and the study designs were miscellaneous. Contributing factors as patient age, body mass index, previous IVF attempts, infertility cause, are all of great significance for the outcome. Furthermore, the protocol type used in the IVF cycle potentially may influence the outcomes, due to the well-known impact on thyroid function, and the data on fertilization method are frequently imprecise. Last, the use of ICSI for male factor infertility and the number of embryos transferred could affect the outcomes. Thus, all these factors may have influenced the interpretation of the results, making the final medical decision often challenging to make.

##### Impact on pregnancy and obstetric outcomes

Overt hypothyroidism is associated with increased risk of hypertension and preeclampsia, abruptio placentae, miscarriage, preterm delivery, postpartum hemorrhage, low birth weight, neonatal respiratory distress, and stillbirth [43]. In case of not adequately treated overt hypothyroidism, an estimated 60% risk of fetal loss occurs [44], together with possible stunted intrauterine growth and mild deficits in neurodevelopment [45]. When happening in early gestational age, changes in behavior and decreased cognitive abilities in the offspring may arise, as well as delayed psychomotor development and impairment in the intellectual development of the offspring [44, 46, 47].

A subclinical form of hypothyroidism has been reported in some studies, depending on a cutoff for TSH when defining subclinical hypothyroidism, to be associated with an increased risk of miscarriage, premature delivery, preeclampsia, and increased fetal mortality, and impairment in neuropsychological tests and vision development of the children [35]. Some studies highlighted a significantly lower miscarriage rate in women with TSH below 2.5 mU/L compared to those with one over 2.5 [48, 49]. Casey et al. conducted a study in a large cohort of 17,298 pregnant women and found that subclinical hypothyroidism (TSH > 3) was associated with an increased risk of premature delivery (before 34 weeks) [50]. These results were not confirmed by Cleary-Goldman et al., who demonstrated no association between higher TSH levels with prematurity (before 37 weeks) [51].

##### Impact on spontaneous conception

Dosiou et al. showed the presence of TPO expression on mature granulosa cells [4]. Three hypotheses are proposed to clarify the connection between TAI and impaired fertility. The first suggests that TAI represents a general autoimmune response, enhancing natural cytotoxicity; the second is that TAI directly affect ovarian tissue; the third indicates that TAI, inducing thyroid function deterioration to overt hypothyroidism, affects reproductive health [4]. Dosiou et al. presented one more model: at early stages, the autoimmunity affects the ovary, when levothyroxine has no impact on outcomes, and antioxidants and immunomodulators as well as inositol may prove instead useful [52, 53]. During the progression of TAI, impaired thyroid response to hCG stimulation arises; this deterioration of thyroid function leads to a further impossibility to adapt it to the increased demands during pregnancy leading to a vicious circle [4]. As a matter of fact, TAI, regardless of thyroid function, is associated with unexplained subfertility [54], and it was suggested that it may represent an important cause of infertility and low ovarian reserve. Confirming this, low antral follicle count, considered a marker of ovarian reserve, and high TPOAb are seen in cases of unexplained infertility [55].

##### Impact on ART outcomes

Different original articles and systematic reviews focused on the relationship between TAI and ART outcomes; however, findings are controversial. A meta-analysis conducted on 1098 subfertile women undergoing IVF (141 with TAI and 957 without TAI) found that the presence of TAI was associated with twofold higher risk of miscarriage, with no significant effect on clinical pregnancy and delivery rates [56], findings confirmed by more recent and larger meta-analyses which also found increased preterm delivery rate [57], and decreased rate of live birth [58].

To this regard, Thangaratinam et al. [57] reported an odds ratio (OR) of miscarriage of 3.15 while Busnelli et al. an OR of 1.44 [58]. Interestingly, the most recent meta-analyses found no difference in ART outcomes [59–61]. Poppe et al. in 2018 investigated the impact of TAI on pregnancy outcomes in infertile women undergoing ICSI treatment, excluding IVF or IUI cycles, accounting for 4 studies for a total of 1855 ICSI cycles (of them, 290 with TAI) reporting no increased risk of early miscarriage [62]. A meta-analysis investigating the effect of levothyroxine treatment in TAI-positive women undergoing ART denied any positive impact on that the miscarriage rate; however, levothyroxine decreased the miscarriage rate if subclinical hypothyroidism was present [63] suggesting that this condition per se may be detrimental [9, 64]. Specific information regarding the effect of thyroid function on controlled ovarian hyperstimulation (COH) is limited, mainly due to the absence of randomized controlled trials.

##### Impact on pregnancy and obstetric outcomes

Stagnaro-Green for the first time showed an association between pregnancy loss and thyroid autoimmunity, thus the patients who were positive for TAI demonstrated a twofold increased risk of pregnancy loss [65], data later confirmed by several other studies and meta-analyses [66–70]. Women with normal thyroid function positive for TPOAb or TgAb also seem to have a significantly higher risk of preterm birth, reaching an OR as high as 2.9, as reported by several large studies and meta-analyses [57, 71–74]. However, it should be noted that three recent large prospective cohort studies showed no significant associations between TAI and risk for premature delivery [75–77]. Increased risk of other complications in TAI-positive women was reported, such as perinatal death [77], placental abruption [78], and postpartum depression [79].

A significant number of studies have evaluated the neurodevelopment outcomes associated with TAI in children of TAI-positive women. Lower motor and intellectual development in the offspring [80] and sensorineural hearing loss were noticed [81]. Interestingly, Williams et al. reported lower perceptual performance and motor scores in children conceived by TgAb-positive mothers, and lower perceptual performance scores in children with TgAb-positive cord blood [82].

There are a growing number of studies trying to elucidate if levothyroxine treatment reduces the risk of maternal and fetal complications with conflicting results. Two randomized studies showed a significant reduction in miscarriages with levothyroxine treatment [83, 84], while one other, very large, did not find any significant difference in live birth rates upon treatment with a fixed dose of 50 mcg levothyroxine started before pregnancy [85]. Finally, one study noted a 69% reduction of preterm birth [57], results confirmed by Negro et al. who showed that thyroid hormone replacement reduced both miscarriage and preterm delivery rates in euthyroid women with TAI [83]. Interestingly, the beneficial impact may be only for those conceiving naturally, as the miscarriage rate was unchanged in those undergoing ART [63].

### Treatment recommendations from latest guidelines

#### TAI and hypothyroidism

The recommendations are very clear in treating overt hypothyroidism before and during pregnancy, but when it comes to subclinical hypothyroidism, there is no consensus on whether to treat it or not, and, as previously mentioned, the concept of subclinical hypothyroidism itself is currently a matter of debate. According to the latest ATA guidelines, TPOAb should be measured in all pregnant women with TSH > 2.5 mU/L. All women with a TSH greater than 10.0 mU/L should start treatment even when fT3/fT4 are within ranges. TPOAb+ women should be treated if with a TSH greater than the pregnancy-specific reference range and may be treated if with TSH concentrations > 2.5 mU/L and below the upper limit of the pregnancy-specific reference range. TPOAb− women may be treated if with TSH concentrations greater than the pregnancy-specific reference range and below 10.0 mU/L, but should not be treated with a normal TSH (TSH within the pregnancy-specific reference range or < 4.0 mU/L if unavailable) [9]. TPO/TgAb + euthyroid women should be monitored with TSH serum levels at the time of pregnancy confirmation and every 4 weeks until mid-pregnancy [9]. Both ATA and ETA guidelines confirm that isolated hypothyroxinemia, defined as low fT4 concentrations with TSH in reference ranges, should not be routinely treated in pregnancy [9, 27].

If starting an ART procedure, the latest recommendations suggest measuring TSH and TPO/TgAb in all women. Women with TSH > 4.0 mIU/L should start treatment to TSH < 2.5 mIU/L, and women with TAI and TSH levels between 2.5 and 4 mIU/L could benefit from treatment on a case-by-case basis to optimize embryo development [9, 27]. In TPOAb− women with TSH > 2.5 mIU/L, sonographic criteria of TAI may be sought, although this is operator dependent and should, therefore, be considered with caution [27]. TPOAb− women with a TSH between 2.5 and 4 and no ultrasonographic finding of TAI should not be treated [27] (Table 2).

**Table 2:** Management of thyroid dysfunction during spontaneous conception, ART treatment and pregnancy, according to the latest guidelines

Hyperthyroidism and pregnancy	- In case of hyperthyroidism (increased fT3 and/or fT4), pregnancy (both spontaneous pregnancy and ART treatment) should be planned after normalization of thyroid function -If TSH < 0.3 but normal fT3/fT4, ART may not be postponed -Propylthiouracil is the preferred drug in the first 16 weeks -fT4 levels should be kept in the upper third of the normal non-pregnant reference range, without aiming at TSH normalization		
Hypothyroidism, thyroid autoimmunity and pregnancy		ART procedures	Spontaneous pregnancy
	Should be treated	-All women with overt hypothyroidism -All women with TSH > 4.0 mIU/L	-All women with overt hypothyroidism -All women with TSH > 10 mIU/L -TPOAb+ women with TSH RR–10 mIU/L
	May be treated	-TPOAb+ women with TSH 2.5–4 mIU/L -TPOAb− women with TSH > 2.5 mIU/L and US TAI	-TPOAb+ women with TSH 2.5–RR mIU/L -TPOAb− women with TSH RR–10 mIU/L
	Should not be treated	-TPOAb− women with a TSH 2.5–4 and no US TAI -All women with a TSH < 2.5 -Isolated hypothyroxinemia	-TPOAb− women with a TSH < RR -All women with a TSH < 2.5 -Isolated hypothyroxinemia
Follow-up during pregnancy	- In euthyroid TPO/TgAb+ women TSH concentration should be performed at the time of pregnancy confirmation and every 4 weeks through mid-pregnancy, and at least once near 30 weeks gestation - In women on LT4 treatment undergoing COH, both TAI+/TAI, TSH should be evaluated the day of the confirmatory hCG measurement		

*ART* assisted reproductive technique, *TSH* thyroid-stimulating hormone, *US TAI* ultrasonographic evidence of thyroid autoimmunity, *RR* (pregnancy- and population-specific) reference range, *TPOAb*+ thyroid peroxidase antibodies positive, *TgAb*+ thyroglobulin antibodies positive, *TPOAb− *thyroid peroxidase antibodies negative, *TgAb− *thyroglobulin antibodies negative, *COH* controlled ovarian stimulation

#### Hyperthyroidism

Several options exist to treat hyperthyroidism: radioiodine ablation (RAI), surgical thyroidectomy, or antithyroid drug (ATD) therapy; in pregnancy, the first method is contraindicated, as well as in the 6 months before conception [9]. As RAI was associated with worsening of the ovarian reserve, it is important to adequately inform fertile women, although the scarcity of evidence available does not allow to formulate specific recommendations. Regarding surgery, the optimal time to perform this procedure is during the second trimester, followed by a gradual disappearance of TSH receptor antibodies (TRAb) [86]. Considering ATD, propylthiouracil (PTU) is the preferred drug in the first 16 weeks of pregnancy because of the possibility of teratogenic effects of carbimazole and methimazole (MMI) (aplasia cutis and MMI embryopathy); however, because of the risk of hepatotoxicity in the second half of the gestation, caution is needed [9]. Fetal hyperthyroidism caused by the cross-placental passage of TRAb could appear at or after week 20 of pregnancy, so, a careful monitoring is required and maternal fT4 levels should be kept in the upper third of the normal non-pregnant reference range. Finally, fetal hypothyroidism may also be expected due to overtreatment with the antithyroid drug.

When women seeking ART procedures are found with TSH < 0.3 and increased fT3 and/or fT4, ART should be postponed until an endocrine work up has been conducted. If, however, fT3 and/or fT4 are within ranges, ART may not be postponed [27] (Table 2).

## Thyroid and male fertility

### Physiology

Human testes have two main functions: androgen production and spermatogenesis. Specifically, Leydig cells produce androgenic hormones: testosterone, androstenedione and deidroepiandrosterone, whereas Sertoli cells promote spermatogenesis and release androgen-binding protein (ABP) under FSH stimulation [87]. Thyroid hormones have their nuclear receptors expressed within the testis [88], and influence Sertoli cells, Leydig cells and spermatogenesis through regulation of gene transcription, protein synthesis, proliferation and differentiation [6–8].

Under physiological conditions, T3 inhibits Sertoli cell proliferation and promotes maturation, essential for spermatogenesis [89, 90].

### Pathology

Several endocrine and metabolic diseases are involved in male infertility, such as hypogonadism, diabetes, obesity and adrenal dysfunction [91–95]. Beyond these conditions, thyroid dysfunction may affect male fertility too, albeit this is not widely investigated. Noteworthy, congenital hypothyroidism does not cause impaired development of male reproductive system [96–98], although, on the other hand, if not properly treated with replacement therapy, it causes delayed sexual maturation [96–99], and the treatment of hypo- and hyperthyroidism is associated with an improvement in testis function, but evidence is scarce [89, 100–102].

**Fig. 2:**
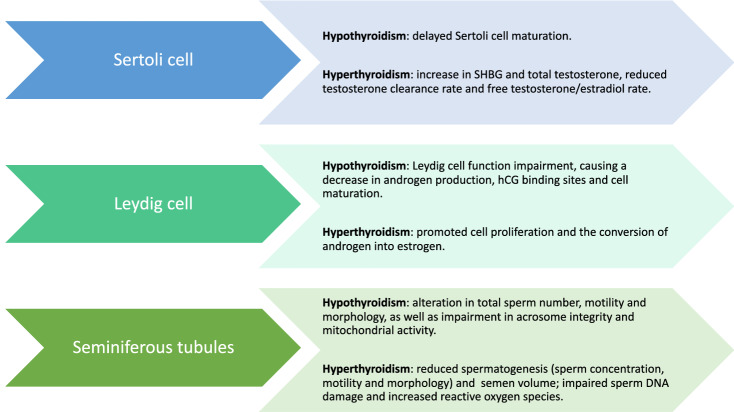
The role of thyroid dysfunction on Sertoli cells, Leydig cells and seminiferous tubules

Patients with primary hypothyroidism show delayed Sertoli cell maturation, with normalization when euthyroidism is restored [103–105], together with Leydig cell function impairment, causing a decrease in androgen production, cell maturation and hCG binding sites [103, 106]. As a consequence, SHBG, total, and free testosterone concentrations are decreased [22] (Fig. 2). Hypothyroidism can also cause an alteration in sperm morphology [22, 107]. Krassas et al. have demonstrated that patients affected by hypothyroidism show more frequently atypical sperm percentage than euthyroid patients [90]. Moreover, they have found a correlation between teratozoospermia and fT4 levels [90], with an improvement in spermatozoa morphology after replacement treatment [90]. Hypothyroidism may also decrease the total sperm number and motility as well as lead to an impairment in acrosome integrity and mitochondrial activity [108], with improved motility upon hormone replacement [89, 109] (Fig. 2).

On the other hand, men with thyrotoxicosis show an increase in SHBG and total testosterone, with normal free testosterone, reduced testosterone clearance rate and free testosterone/estradiol rate, due to elevated total and free estradiol concentration [8, 22, 96, 110–114]. Furthermore, hyperthyroidism promotes the conversion of androgen into estrogen [22, 89] (Fig. 2). According to animal studies, thyrotoxicosis can affect Leydig cells, with delayed cell maturation and spermatogenetic alterations, but promoted cell proliferation [105, 115]. Moreover, acute increase in T3 increases LH receptors on Leydig cells and consequently steroidogenesis, although chronic high levels of T3 have an opposite effect [89]. Conversely, Sertoli cell proliferation is inhibited in hyperthyroidism, with negative effects on spermatogenesis and reduction of testis volume [88, 89]. Hyperthyroidism is also associated with the reduction of sperm concentration, motility and impairment of sperm morphology, as well as a reduction of semen volume [107, 116] (Fig. 2). The effect of hyperthyroidism on semen has been described in different studies. Krassas et al. have seen that the treatment of thyrotoxicosis improves sperm motility, without significant changes in sperm morphology and count [117, 118]. Moreover, high levels of thyroid hormones can cause sperm DNA damage and infertility. Indeed, high levels of T3 and T4 promote an increase in reactive oxygen species (ROS) and consequently oxidative stress [119–121]. Finally, fT4 seminal plasma levels were recently assessed by Condorelli et al. (3.15 ± 0.7 pmol L^−1^) [6]; the authors also evaluated the effect of bio-functional sperm parameters after incubation of semen with increasing concentrations of levothyroxine and found reduced sperm necrosis and lipid peroxidation along with an improvement in chromatin compactness with a levothyroxine concentration of 2.9 pmol L^−1^. This in vitro study could open a new scenario of clinical application in patients with idiopathic infertility, although further studies are warranted to identify thyroid hormone seminal plasma reference ranges possibly representing the appropriate semen-thyroid hormones balance. What is currently well established is that treatment of hyperthyroidism, restoring normal or high-normal level of T4, improves seminal parameters [120, 122].

## Conclusions

Infertility affects millions of people during reproductive age worldwide. Male and female factors on this condition contribute similarly (20–30%), and the endocrine system plays a role in this condition. Among endocrine conditions, thyroid dysfunction is frequently associated with female infertility, with emerging evidence on male fertility, and ART outcomes are influenced as well. Thyroid function should, therefore, be carefully monitored in both male and female, in couples seeking spontaneous pregnancy as well as ART, as treatment is generally immediate and likely to improve chances of success.
